# Structural insights into *Plasmodium* PPIases

**DOI:** 10.3389/fcimb.2022.931635

**Published:** 2022-09-02

**Authors:** Sreekanth Rajan, Ho Sup Yoon

**Affiliations:** ^1^ School of Biological Sciences, Nanyang Technological University, Singapore, Singapore; ^2^ College of Pharmacy, CHA University, Pocheon-si, South Korea; ^3^ CHA Advanced Research Institute, Seongnam-si, South Korea

**Keywords:** malaria, plasmodium, PPIase, FKBP, cyclophilin, FK506, cyclosporin

## Abstract

Malaria is one of the most prevalent infectious diseases posing a serious challenge over the years, mainly owing to the emergence of drug-resistant strains, sparking a need to explore and identify novel protein targets. It is a well-known practice to adopt a chemo-genomics approach towards identifying targets for known drugs, which can unravel a novel mechanism of action to aid in better drug targeting proficiency. Immunosuppressive drugs cyclosporin A, FK506 and rapamycin, were demonstrated to inhibit the growth of the malarial parasite, *Plasmodium falciparum*. Peptidyl prolyl *cis/trans* isomerases (PPIases), comprising cylcophilins and FK506-binding proteins (FKBPs), the specific target of these drugs, were identified in the *Plasmodium* parasite and proposed as an antimalarial drug target. We previously attempted to decipher the structure of these proteins and target them with non-immunosuppressive drugs, predominantly on FKBP35. This review summarizes the structural insights on *Plasmodium* PPIases, their inhibitor complexes and perspectives on drug discovery.

## Introduction

Malaria is one of the world’s most dreadful parasitic diseases affecting millions each year, prevalently in tropical and subtropical regions (Liu et al., 2021; White et al., 2022). This vector-borne disease is caused by a single-celled parasite belonging to the genus *Plasmodium*, transmitted and spread by the infected female *Anopheles* mosquitos. Among the many *Plasmodium* species, five have been identified as responsible for transmitting malaria in humans. *Plasmodium falciparum* (*Pf*) is the most infectious, followed by *P. vivax* (*Pv*), apart from *P. malariae, P. ovale, and P. knowlesi*. Vector control (Van De Straat et al., 2022), chemoprophylaxis (Plowe, 2022), antimalarial drugs (Tse et al., 2019), and, recently, a protein-based vaccine (Laurens, 2020) are the presently adopted methods for treating malaria. The complex life cycle of the *Plasmodium* (**Supplementary Figure 1**) involving the pathogen (parasite), carrier (mosquito), and the host (human) compounded by the emergence of drug-resistant strains (White, 2004; Balikagala et al., 2021), makes it a challenging organism for drug targeting. The most common malaria drug targets are the parasite proteins such as lactate dehydrogenase, plasmepsin2, dihydrofolate reductase, and others (Mehlin, 2005; Yang et al., 2021). Drug repurposing is an efficient starting point for identifying and characterizing novel targets (Pushpakom et al., 2019). In one such chemo-genomics effort immunosuppressive drugs, cyclosporin A (CsA), FK506 and rapamycin were shown to possess antimalarial properties (Bell et al., 1994), leading to the identification of their target PPIases in the parasite.

## PPIases and malaria

CsA, FK506 and rapamycin are well-known high-affinity/specific inhibitors of a family of proteins, peptidyl-prolyl *cis/trans* isomerases (PPIases) comprising of (a) cyclophilins (CyPs), which bind CsA, (b) FK506-binding proteins (FKBPs), which bind FK506 and rapamycin, and (c) parvulins (Galat, 2003; Harikishore and Yoon, 2015). As the name indicates, PPIases primarily function as chaperone proteins, enabling the conversion of *cis* to *trans* configuration of proline, a rate-limiting step in protein folding. Apart from inhibiting the PPIase activity, CsA/FK506 acts as an intermolecular glue between CyP/FKBP and calcineurin (CaN), a Ca^2+^/calmodulin (CaM)-dependent protein serine/threonine phosphatase, thereby inhibiting CaN activity. This signaling leads to a cascade of downstream events resulting in immunosuppression. On the other hand, rapamycin binds to FKBPs to bridge its interaction with the mammalian target of rapamycin (mTOR), also resulting in immunosuppression. Therefore, PPIases are also called immunophilins, due to the immunosuppressive action exerted by these molecules. While these ligands have been primarily used to treat rejection during organ transplant, they have also been repurposed for eczema, cancer (Hasskarl, 2018) and other clinical applications.

Angus Bell and coworkers showed that CsA, FK506 and rapamycin (Bell et al., 1994) could be repurposed for malaria, as they exhibited *in vitro P. falciparum* inhibition. Further they also showed that the non-immunosuppressive analogs of FK506 (Monaghan et al., 2005) and CsA (Bell et al., 1996) also have the similar effects, which was considered a promising sign toward drug discovery.

A search of *Plasmodium* PPIases in the PlasmoDB (Amos et al., 2022) revealed the presence of 2 FKBPs and 11 CyPs (**Supplementary Table 1**). All of them possess a PPIase catalytic domain, apart from other domains such as Tetratricopeptide repeat (TPR), CaM, indicating that these PPIases may act as chaperones or be part of a multi-protein complex (**Supplementary Figure 2**). Both PPIases were characterized for their (a) PPIase, (b) CaN inhibition, and (c) chaperone activities to decipher their probable function (Gavigan et al., 2003; Monaghan and Bell, 2005; Bell et al., 2006; Yoon et al., 2007; Marin-Menendez et al., 2012).

## *Plasmodium* cyclophilins

CsA and its non-immunosuppressive analogs inhibit the intraerythrocytic growth of *P. facliparum*. An affinity pulldown on *P. facliparum* proteins using CsA identified two cyclophilins, *Pf*CyP19A and *Pf*CyP19B, constituting ~1.2% and ~0.5% of the parasite protein, respectively. The mRNA levels of *Pf*CyP19A, *Pf*CyP19B, and *Pf*CyP24, revealed that they are abundant in the intraerythrocytic cytosol (Hirtzlin et al., 1995; Reddy, 1995; Le Roch et al., 2003), predominantly in the middle of the erythrocytic cycle, late trophozoite/schizont and immature (ring) stages (Gavigan et al., 2003; Bell et al., 2006), respectively. Apart from the above, a few other *Pf*CyPs were also identified (Marin-Menendez and Bell, 2011; Marin-Menendez et al., 2012). Sequence comparison of the CsA-interacting residues of human (*hs)*CyPs with *Pf*CyPs revealed minor variation (Marin-Menendez et al., 2012). The highly conserved Trp121 (*hs*CyPA-numbering) is found in six of 11 *Plasmodium* CyPs. Most of the other residues within 4Å from CsA are also mostly conserved among *Pf*CyPs, with exceptions in *p*CyP72 and *p*CyP81 (**Supplementary Table 2**). It was shown that only *Pf*CyP19A and *Pf*CyP19B possessed PPIase activity which was inhibited by CsA with an IC_50_ of 10nM and 15nM, respectively (Marin-Menendez et al., 2012). Nonetheless, all *Pf*CyPs exhibited chaperone activity, by preventing the aggregation of the model substrate, rhodanese and a few against citrate synthase (Marin-Menendez et al., 2012). The CaN inhibition of *Pf*CyP19 was observed in the presence of CsA (Bell et al., 1994; Gavigan et al., 2003), similar to the *hs*CyP. Thus, one can conclude that *Pf*CyP19 could be a good target for drug discovery. To this end, the crystal structure of *Pf*CyP19A in complex with CsA (**Supplementary Figure 3A**), adopts a canonical CyP-fold mainly made up of eight β-strands and two α-helices (Peterson et al., 2000), and comparison revealed negligible differences with its human counterpart (**Supplementary Figure 3B**). The CsA-interacting residues in *Pf*CyPA are identical to those in *hs*CyPs, and so are their interactions. Owing to the highly hydrophobic active site, a few non-classical C-H … O hydrogen bonds (Rajan et al., 2013), apart from the hydrogen-bonded and non-polar interactions, were observed (Peterson et al., 2000), which are also conserved in the human counterpart (**Supplementary Table 3**). In addition, a serine protease-like catalytic triad was identified near the active site, but their mutation did not alter the enzyme’s catalytic properties. Thus, it was concluded that generating specific inhibitors for *Pf*CyPA would be difficult, owing to the indistinguishable active site residues and interactions. We tried to compare the CsA-interacting residues within 4-5Å to identify any residue differences and observed that Thr63 (*hs*CyPA numbering) mutated to Ser; this could be considered a starting point toward specificity (**Supplementary Table 2**). Outside the active site region, additional six residues are inserted in the loop between α1 and β3, observed in *Pf*Cyp19A and *Py*CyP24 but not in *Py*CyP23 and *Pf*CyP87 (**Supplementary Figure 3C**). The functional relevance, if any, of this insertion still needs to be deciphered and could play a role in their localization or chaperone function.

## Plasmodium FKBPs

Extensive studies were done on *Pf*FKBPs compared to *Pf*CyPs for reasons mentioned herein. The 35-kDa FKBP (Braun et al., 2003) was the first identified FKBP in *P. falciparum* (*Pf*FKBP35), and later highly similar homologues in the other malarial parasites, *P. vivax* and *P. knowlesi*, were also observed (Cui et al., 2005; Hall et al., 2005) (**Supplementary Table 1**). *Pf*FKBP35 is expressed throughout the intraerythrocytic life cycle (Kumar et al., 2005a), predominantly in the cytosol during the ring stage. However, it can translocate into the nucleus in the trophozoites and schizonts stages. *Pf*FKBP35’s localization in the nucleus could be due to the presence of the TPR domain (Alag et al., 2009a) (**Supplementary Figure 2**), which interacts with *Pf*Hsp90 (Kumar et al., 2005a; Kumar et al., 2005b). Functionally, FK506 showed an IC_50_ of 260nM and 160nM against the PPIase activity of *Pf* FK506 binding domain35 (*Pf*FKBD35) and *Pv*FKBD35, respectively (Alag et al., 2010), while rapamycin’s IC_50_ is 480nM against *Pf*FKBD35 (Monaghan and Bell, 2005). In addition, *Pf*FKBP35 also exhibited chaperone activity against model substrates, rhodanese, and citrate synthase. Although FK506 did not inhibit the activity of full-length *Pf*FKBP35 or TPR domain alone, it affected *Pf*FKBD35’s PPIase activity, suggesting that *Pf*FKBD35 has a role in *Pf*FKBP35’s chaperone activity (Monaghan and Bell, 2005). Earlier studies reported that FKBP35 binds to CaN in the absence of FK506 (Kumar et al., 2005b; Monaghan and Bell, 2005; Monaghan et al., 2017). On the other hand, our studies showed that this molecular interaction is negligible in the absence of FK506, which was enhanced when FK506 was added (Yoon et al., 2007). Future studies may help validate the discrepancy.

**Figure 1:**
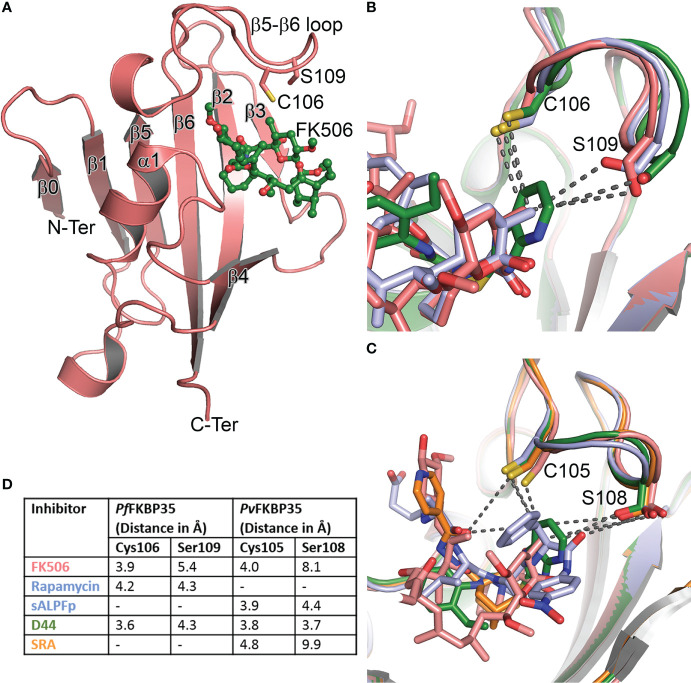
Structure and comparison of *Plasmodium* FKBPs. **(A)** The cartoon representation of *Pf*FKBD35 in complex with FK506 (in ball and stick mode). The β0 observed here is not present in *hs*FKBPs. Similarly, the two unique residues pertaining to *Plasmodium* FKBP35’s Cys106 and Ser109 in the β5-β6 loop are shown in stick mode. **(B)** The overlay of FK506 (salmon), rapamycin (pale blue) and D44 (green) bound structures of *Pf*FKBD35, with the nearest atom from Cys106 and Ser109 shown in broken lines. **(C)** Similarly, the overlay of FK506 (salmon), substrate-ALPF (pale blue), D44 (green) and SRA (orange) bound structures of *Pv*FKBD35. **(D)** The nearest distance between the ligands and the unique Cys and Ser residues are listed for comparison, indicating the D44 is oriented closer to these residues.

While the above observations promised to coin *Pf*FKBP35 as a probable drug target, it is the two unique active site residues identified from the sequence-structure comparison that made it an attractive target. The *Pf/Pv*FKBD35 structures in apo and FK506-bound forms were determined using NMR and crystallography (Kang et al., 2008; Kotaka et al., 2008; Alag et al., 2009b; Alag et al., 2010), respectively. The structure adopts a typical FKBP fold, consisting of a six stranded β-sheet and a short α-helix, with an additional β-strand at the N-terminus (**Figure 1A**). We also successfully determined the substrate-bound structure of FKBP, the first of its kind to our knowledge, in which the critical proline residue of the substrate (ALPF) adopted a *cis* configuration, overlaying on the pipecolinyl moiety of FK506 (Alag et al., 2013). A comparison of the FK506 interacting residues in *hs*FKBP12 with *Plasmodium* FKBD35 indicated that most of them are identical, except for Q53G, H87C, and I90S (*hs*FKBP12 numbering) (**Supplementary Table 4**). Of these, the sidechain atoms of Cys106 and Ser109 in the β5-β6 loop of *Pf*FKBD35 were located at a distance of 3.9Å and 5.4Å from the pyranose group of FK506 (**Figures 1B, D**). A similar observation was also made on the *Pv*FKBD35-FK506 complex, with residues Cys105 and Ser108 (**Figures 1C, D**). Sequence comparison with all *hs*FKBPs also revealed that these two residues are unique to *Plasmodium* FKBP35s (**Supplementary Table 4**), which is promising as it helps to address the specificity towards the parasite inhibition. To support this, the estimated lower physiological levels of *Pf*FKBP35 (0.05-0.1μM) in the parasite in comparison to *hs*FKBP12 (3-5μM) (Braun et al., 2003) also aid in achieving this, provided we design non-immunosuppressive inhibitors with higher affinity towards Cys106 and Ser109. With this aim, an *in silico* pharmacophore approach was adopted to identify small molecules targeting these two residues (Harikishore et al., 2013b). This resulted in a molecule containing purine-like and phenyl-ethyl ring systems linked by a thioacetamide linker, labeled as D44 (**Supplementary Table 5**). The IC_50_ of D44 against the PPIase activity of *Pf*FKBD35 and *Pv*FKBD35 was shown to be 132nM and 125nM, respectively. D44 also exhibited *in vitro* inhibition of *P. falciparum* 3D7 intraerythrocytic growth with an IC_50_ of 234nM. It arrested the parasite growth at the schizont stage, similar to FK506 (Harikishore et al., 2013b). D44 is a non-immunosuppressive FKBP35 inhibitor, as observed from the calcineurin phosphatase assay. Based on the above results, we studied the molecular binding of D44 with *Pf/Pv*FKBD35 using NMR titration and X-ray crystallography. NMR revealed that D44 perturbs the essential active site residues, while the co-crystal structures revealed the precise mode of interactions. A few critical active site residues, especially Trp78, swings inwards in the D44-bound structure to compensate the bulkier pipecolinyl moiety in FK506 (**Supplementary Figure 4**). More importantly, the purine-like ring in D44 can be seen oriented towards Cys106/105 and Ser109/108 in *Pf/Pv*FKBD35, at a distance of 3.6/3.8Å and 4.3/3.7Å, respectively, compared to 3.9/4.0Å and 5.4/8.1Å in the FK506-bound form (**Figure 1D**). The hydroxyl sidechain group of Ser108 flips towards the purine-like ring of D44 in the *Pv*FKBD35-D44 structure, probably owing to its better resolution. Alternately, we also identified another inhibitor using a ligand-based approach in which adamantyl and pyridine rings were connected by a carbonyl hydrazide linker, which we labelled SRA (Harikishore et al., 2013a) (**Supplementary Table 5**). This small molecule inhibited the PPIase activity of *Pf*FKBD35 and *Pv*FKBD35, with an IC_50_ of 83nM and 75nM, respectively, and did not impinge on calcineurin, similar to D44. *in vitro* parasite inhibition revealed that SRA acts on the trophozoite stage of parasite growth, with an IC_50_ of 250nM. The crystal structure of *Pv*FKBD35 bound to SRA revealed that the adamantyl and pyridine rings of SRA overlay on to the pipecolinyl and cyclohexyl rings of FK506, respectively. The four-atom linker is stabilized by hydrogen bonds to orient the pyridine ring. The key here is the anchor-like interaction made by the bulky adamantyl moiety, which occupies the base of the active site pocket. It was evident that both these small molecules inhibited the parasite’s growth in the intraerythrocytic stages, where FKBP35 expresses abundantly (**Supplementary Figure 1**). Recently, another small molecule, [4.3.1]-aza-bicyclic sulfonamide ([4.3.1]-ABS) (**Supplementary Table 5**), possessed an enzymatic IC_50_ of 98nM and inhibited the *P. falciparum* growth with an IC_50_ of 2.3μM (Pomplun et al., 2018) (**Table 1**).

**Table 1:** List of *Plasmodium* PPIases, their inhibitors, IC_50,_ and PDB ID, if available.

Protein Name	Ligand	IC_50_ in μM‡	PDB ID	RMSD in Å (No. of atoms)
*Pf*FKBD35	FK506	1.9	2VN1 (Kotaka et al., 2008)	0.58 (77)!
	Rapamycin	2.6	4QT2 (Bianchin et al., 2015)	0.55 (73)
	Rapamycin		4QT3 (Bianchin et al., 2015)	0.51 (65)
	D44	0.235	4J4N (Harikishore et al., 2013b)	0.60 (78)
	SLF-covalent analogs	1.4-1.9	NA (Atack et al., 2020)	–
	[4.3.1.]ABS	2.3	NA (Pomplun et al., 2018)	–
	–		2OFN* (Kang et al., 2008)	1.89 (73)
*Pv*FKBD35	FK506		3IHZ (Alag et al., 2010)	0.54 (72)
	sALPFp		4ITZ (Alag et al., 2013)	0.39 (71)
	D44		4J4O (Harikishore et al., 2013b)	0.44 (71)
	SRA	0.25	4MGV (Harikishore et al., 2013a)	0.48 (77)
	–		3NI6 (Alag et al., 2013)	0.43 (69)
	–		2KI3* (Alag et al., 2009b)	1.23 (86)
*Pv*FKBP25	–		4JYS (Rajan et al., 2014)	1.47 (71)
*Pf*CyP19A	Cyclosporin	0.30	1QNG (Peterson et al., 2000)	0.32 (133)!!
	Cyclosporin		1QNH^#^ (Peterson et al., 2000)	0.27 (136)
*Py*CyP23	–		2B71 (Vedadi et al., 2007)	0.51 (103)
*Py*CyP24	–		1Z81 (Vedadi et al., 2007)	0.61 (146)
*Pf*CyP87	–		2FU0 (Vedadi et al., 2007)	0.80 (107)

The RMSD of these structures are also provided.

‡in vitro P. falciparum inhibition; *Determined by NMR, all others are crystal structures.

^#^mutant form; Pf, P. falciparum; Pv, P. vivax; Py, P. yoelii;!-against hsFKBP12 (PDB ID:1PPN);!! against hsCyPA (PDB ID:1CWA); NA, Not available.

In all these crystal structures (**Table 1**), the FKBDs form a crystallographic dimer, either in the asymmetric unit or by crystal packing, primarily owing to the Cys105/106, which forms a disulfide bond with the same cysteine from the neighboring chain, except in *Pf*FKBD35-FK506 complex, with the active site from each chain facing each other. Interestingly, the FKBD35 remains a monomer in solution, whereas the full-length FKBP35 is present as a dimer (Yoon et al., 2007), indicating that the dimer formation is due to TPR domain and not FKBD. Therefore, the observed dimer and disulfide bond could have resulted from the crystallization conditions and incubation time for crystal formation. The crystal structures showed that this is a reactive cysteine and being a unique residue of the *Plasmodium* FKBPs, it was proposed to be an attractive site for designing covalent inhibitors which can enhance the specificity of *Plasmodium* FKBP35 (Kotaka et al., 2008). Toward this end, the rapamycin-bound *Pf*FKBD35 was determined in the oxidized and reduced forms (Bianchin et al., 2015). Monomeric units were captured in the crystal. In the oxidized form the Cys106 could be seen covalently attached to dithiothreitol and did not affect the rapamycin interactions. Moreover, the nearest distance from the sidechains of Cys106 and Ser109 to rapamycin is 4.2Å and 4.3Å, respectively (**Figure 1D**).

Recently, another study showed that the modification of D44 terminal phenyl ethyl with covalent warheads were not promising. In contrast, synthetic ligand for FKBP (SLF) analogs (**Supplementary Table 5**) were shown to covalently bind to FKBP35 (Atack et al., 2020), possessing an *in vitro* IC_50_ of 1.4 – 1.9μM against *P. falciparum* (**Table 1**). It was observed that the covalent bond formation is a slow reaction and requires stronger electrophilic warheads, corroborating with our solution studies (Yoon et al., 2007) where it remained a monomer and disulfide bond formation was observed only under the crystal conditions, probably owing to the longer time required for crystal formation. Thus, targeted covalent inhibition of FKBP35 has been considered the way forward (Macdonald and Boyd, 2015).

Another annotated *Plasmodium* FKBP, a 25kDa *Pv*FKBP25, was also characterized by our group (Rajan et al., 2014). The domain architecture indicated the presence of an N-terminal Helix-Loop-Helix domain, followed by a FKBD and a C-terminal CaM-binding domain (**Supplementary Figure 2**), similar to the *hsF*KBP25 (Prakash et al., 2016). *HsFKBP25* is a unique member of the FKBP family possessing multiple binding partners. Biochemical characterization of *Pv*FKBP25 exposed its noncanonical nature lacking catalytic activity, owing to the active site mutations (**Supplementary Table 4**). Its structure unveiled the concealed active site, which is unconducive for ligand binding to validate the noncanonical function of this protein (**Supplementary Figure 5**). Thus, it would be interesting to decipher the role of this protein in the parasite life cycle.

## Conclusion

11 *Plasmodium* CyPs and 2 *Plasmodium* FKBPs have been identified, while only one in each, CyP19 and FKBP35, possess catalytic activity. The question remains as to the function of other PPIases and why the parasite needs multiple PPIases. The possible role of these PPIases in liver stage (Derbyshire et al., 2012; Fontinha et al., 2020) can be explored, though most of them are abundantly produced in the intraerythrocytic stage. Their inhibitors also act on stages which correlate with this (**Supplementary Figure 1**). *Pf*CyP19, only when in complex with CsA, can inhibit CaN, unlike the conundrum revolving the role of FKBP35/FK506 and CaN. The parasite possesses a CaN but lacks an mTOR, the targets of FK506 and rapamycin, respectively. Both inhibitors affect the parasite growth, similarly, indicating that FKBP35 is the target of interest. Although these inhibitors have an affinity for *Plasmodium* FKBP35, they bind stronger with host FKBPs which remains to be a challenge for targeting. On the other hand, if it is true that *Plasmodium*FKBP35 can bind CaN sans FK506, it could be one of the parasite’s arsenal to enhance immunosuppression to hijack the host during infection (Calle et al., 2021). Studies have shown that *Plasmodium* CaN is involved in the erythrocyte invasion by the parasite and regulated host-cell interaction (Paul et al., 2015; Philip and Waters, 2015), which is also intriguing. Thus, a few open questions still lure around FKBP35’s precise function in the parasite and their relationship with CaN, warranting further investigation. Identifying *Plasmodium* PPIase’s natural substrates and characterizing the binding proteins (Leneghan and Bell, 2015) may also help the cause to understand their functional relevance as a chaperone. From a structural perspective, the role of an additional β-strand (β0) (**Figure 1A**) at the N-terminal of *Plasmodium*FKBP35, and insertion in the loop between α1 and β3 of *Pf*CyP19A/*Py*CyP24 (**Supplementary Figure 3C**) is also inquisitive. In future, the structures (**Table 1**) and the interactions (**Supplementary Tables 3, 6**) made by these inhibitors (**Supplementary Table 5**) can aid the design of more specific inhibitors. In addition, the D44’s purine-like ring can be linked with the adamantyl ring of SRA, which could improve its affinity for FKBP35 or covalent warheads can be attached to the purine-like moiety closer to Cys106. The promising [4.3.1.]ABS analogs can also be modified as potential covalent inhibitors. Nonetheless, its inhibition still would be beneficial, especially with the help of non-immunosuppressive covalent inhibitors.

## Author contributions

SR and HY wrote the manuscript and approved the submitted version.

## Conflict of interest

The authors declare that the research was conducted in the absence of any commercial or financial relationships that could be construed as a potential conflict of interest.

## Publisher’s note

All claims expressed in this article are solely those of the authors and do not necessarily represent those of their affiliated organizations, or those of the publisher, the editors and the reviewers. Any product that may be evaluated in this article, or claim that may be made by its manufacturer, is not guaranteed or endorsed by the publisher.
