# Supplementary material for: Structural insights into Plasmodium PPIases
Source: Front Cell Infect Microbiol. 2022 Sep 2;12:931635. doi: 10.3389/fcimb.2022.931635 (PMC9478106; doi:10.3389/fcimb.2022.931635)
Supplement: Supplementary file 1 [file Presentation_1.pptx]

## Slide 1
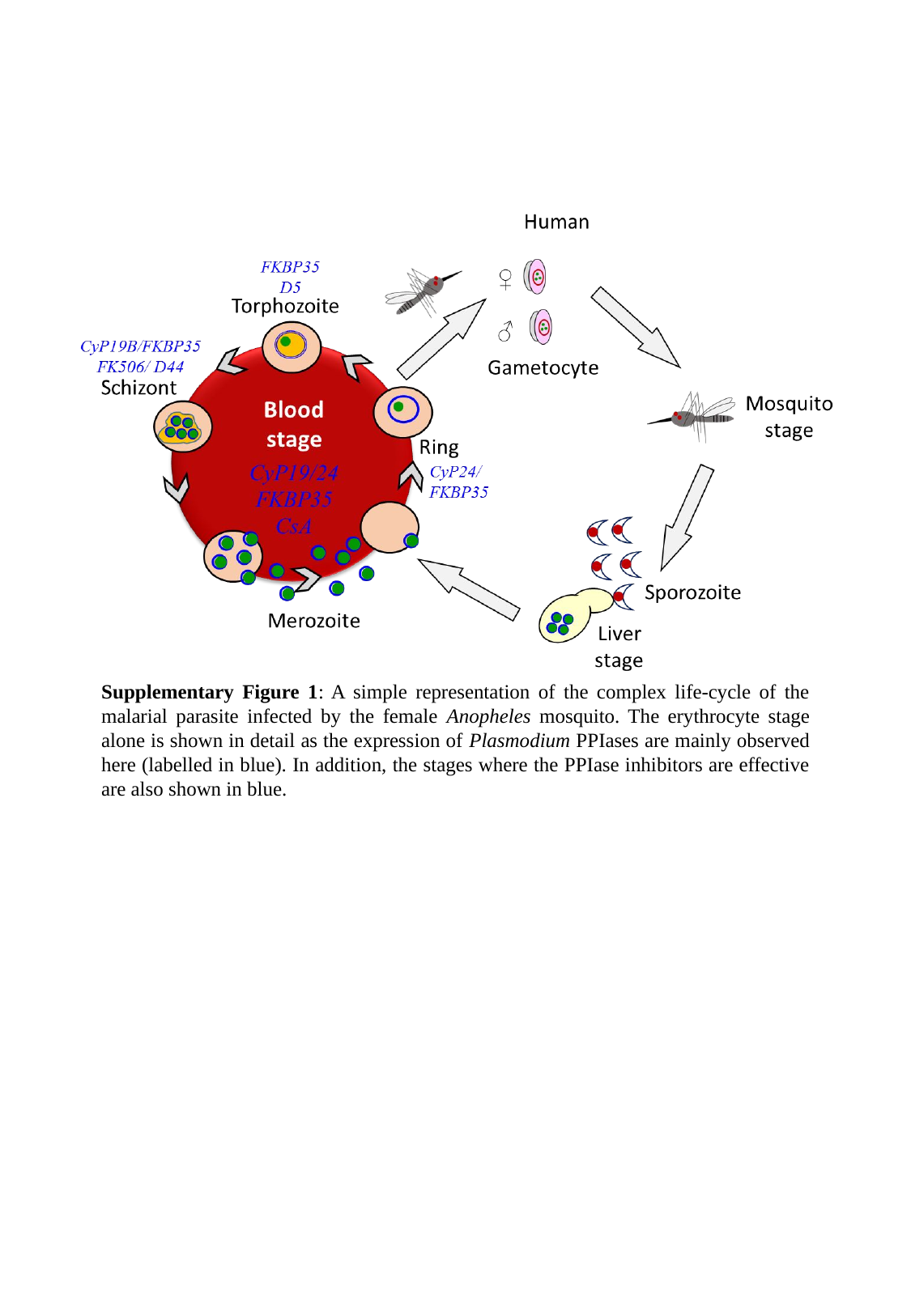

Supplementary Figure 1: A simple representation of the complex life-cycle of the malarial parasite infected by the female Anopheles mosquito. The erythrocyte stage alone is shown in detail as the expression of Plasmodium PPIases are mainly observed here (labelled in blue). In addition, the stages where the PPIase inhibitors are effective are also shown in blue.

## Slide 2
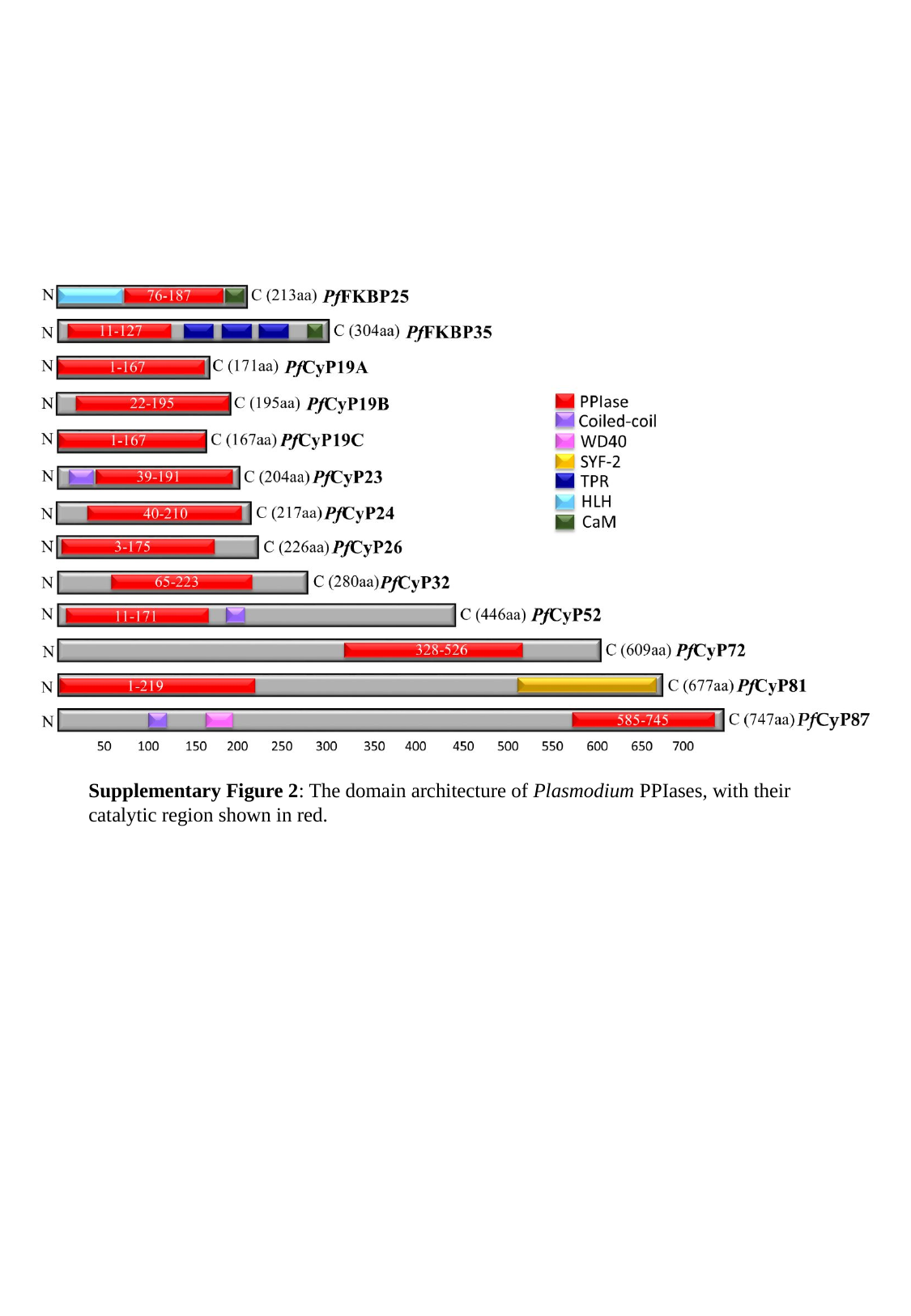

Supplementary Figure 2: The domain architecture of Plasmodium PPIases, with their catalytic region shown in red.

## Slide 3
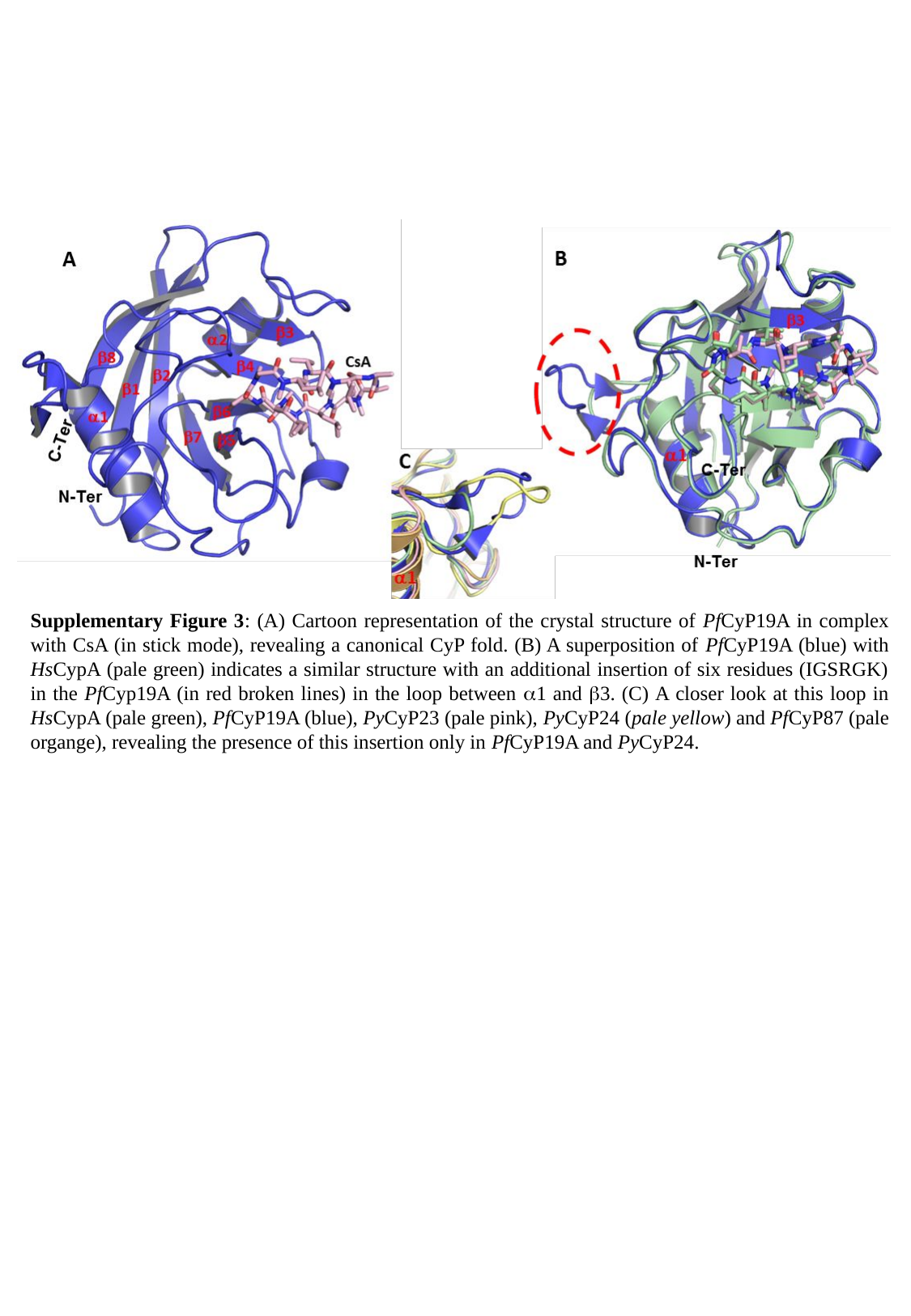

Supplementary Figure 3: (A) Cartoon representation of the crystal structure of PfCyP19A in complex with CsA (in stick mode), revealing a canonical CyP fold. (B) A superposition of PfCyP19A (blue) with HsCypA (pale green) indicates a similar structure with an additional insertion of six residues (IGSRGK) in the PfCyp19A (in red broken lines) in the loop between α1 and β3. (C) A closer look at this loop in HsCypA (pale green), PfCyP19A (blue), PyCyP23 (pale pink), PyCyP24 (pale yellow) and PfCyP87 (pale organge), revealing the presence of this insertion only in PfCyP19A and PyCyP24.

## Slide 4
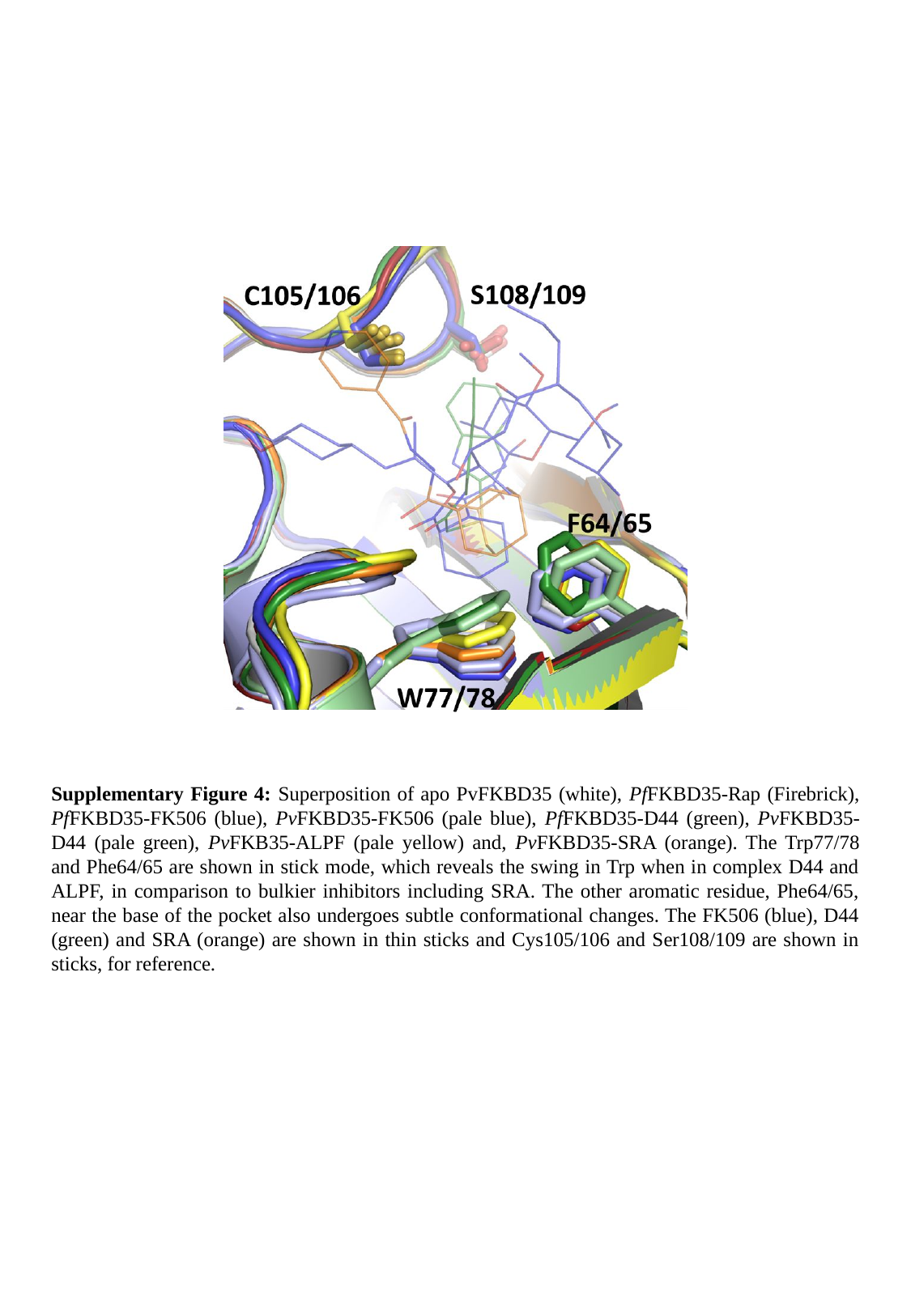

Supplementary Figure 4: Superposition of apo PvFKBD35 (white), PfFKBD35-Rap (Firebrick), PfFKBD35-FK506 (blue), PvFKBD35-FK506 (pale blue), PfFKBD35-D44 (green), PvFKBD35-D44 (pale green), PvFKB35-ALPF (pale yellow) and, PvFKBD35-SRA (orange). The Trp77/78 and Phe64/65 are shown in stick mode, which reveals the swing in Trp when in complex D44 and ALPF, in comparison to bulkier inhibitors including SRA. The other aromatic residue, Phe64/65, near the base of the pocket also undergoes subtle conformational changes. The FK506 (blue), D44 (green) and SRA (orange) are shown in thin sticks and Cys105/106 and Ser108/109 are shown in sticks, for reference.

## Slide 5
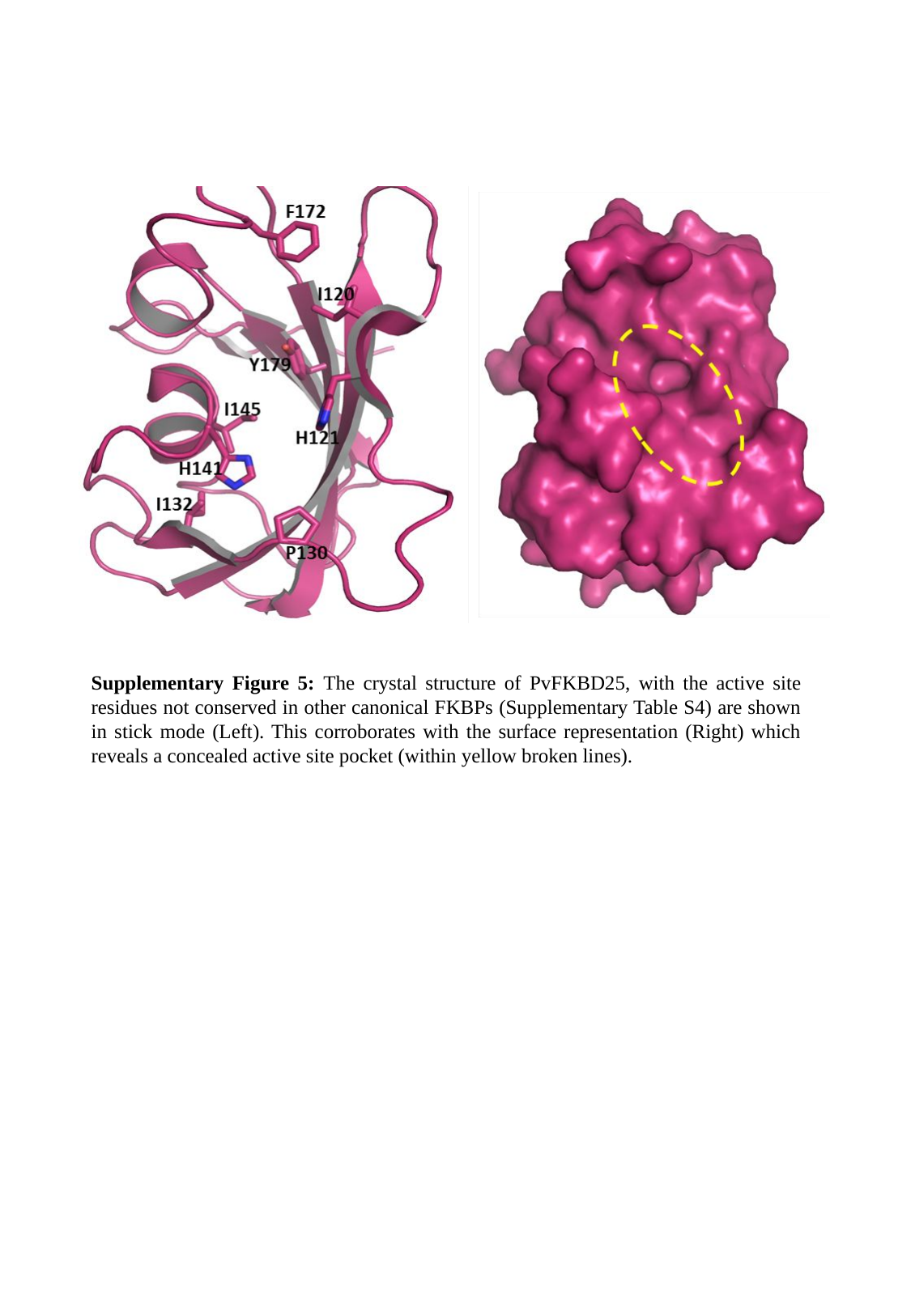

Supplementary Figure 5: The crystal structure of PvFKBD25, with the active site residues not conserved in other canonical FKBPs (Supplementary Table S4) are shown in stick mode (Left). This corroborates with the surface representation (Right) which reveals a concealed active site pocket (within yellow broken lines).
