# Supplementary material for: Structural insights into Plasmodium PPIases
Source: Front Cell Infect Microbiol. 2022 Sep 2;12:931635. doi: 10.3389/fcimb.2022.931635 (PMC9478106; doi:10.3389/fcimb.2022.931635)
Supplement: Supplementary file 2 [file Table_1.docx]

**Supplementary Table 1**: List and details of PPIases in the malarial parasites *Plasmodium falciparum* (*Pf*); *Plasmodium vivax* (*Pv*); *Plasmodium knowlesi* (*Pk*) annotated from PlasmoDB

| S.No. | PPIase | Source-Strain | PlasmoDB  ID | UniProt  ID | No. of amino acids | MW (kDa) |
| --- | --- | --- | --- | --- | --- | --- |
| FKBP | | | | | | |
| 1 | FKBP25 | *Pf*-3D7 | PF3D7_1313300 | C0H5B2 | 213 | 25.6 |
|  |  | *Pv*-Sal1 | PVX_122487 | A5JZC2 | 215 | 25.2 |
|  |  | *Pk*-H | PKNH_1413900 | A0A1Y3DI60 | 216 | 25.4 |
| 2 | FKBP35 | *Pf*-3D7 | PF3D7_1247400 | Q8I4V8 | 304 | 34.8 |
|  |  | *Pv*-P01 | PVP01_1464500 | A5K8X6 | 302 | 34.0 |
|  |  | *Pk*-H | PKNH_1467100 | A0A1Y3DHI1 | 302 | 34.6 |
| Cyclophilin | | | | | | |
| 1 | CYP19A | *Pf*-3D7 | PF3D7_0322000 | Q25756 | 171 | 19.0 |
|  |  | *Pv*-P01 | PVP01_0818200 | A0A1G4HBM6 | 170 | 18.8 |
|  |  | *Pk*-H | PKNH_0818800 | A0A1Y3DMW7 | 170 | 18.7 |
| 2 | CYP19B | *Pf*-3D7 | PF3D7_1115600 | Q27745 | 195 | 21.7 |
| 3 | CYP19C | *Pf*-3D7 | PF3D7_1116300 | Q8IIK3 | 167 | 18.6 |
|  |  | *Pv*-P01 | PVP01_0916900 | A0A1G4HCW7 | 167 | 18.5 |
|  |  | *Pk*-H | PKNH_0914000 | A0A1Y3DNW9 | 167 | 18.5 |
| 4 | CYP23 | *Pf*-3D7 | PF3D7_0528700 | Q8I3I0 | 204 | 23.2 |
|  |  | *Pv*-P01 | PVP01_1005100 | A0A1G4HDR7 | 203 | 22.9 |
|  |  | *Pk*-H | PKNH_1004000 | A0A1Y3DUS8 | 204 | 23.1 |
| 5 | CYP24 | *Pf*-3D7 | PF3D7_0804800 | Q27716 | 217 | 25.0 |
|  |  | *Pv*-P01 | PVP01_0115700 | A0A1G4GR33 | 262 | 29.0 |
|  |  | *Pk*-H | PKNH_0114400 | A0A1Y3DWZ3 | 250 | 28.1 |
| 6 | CYP26 | *Pf*-3D7 | PF3D7_1202400 | Q8I621 | 226 | 26.5 |
|  |  | *Pv*-P01 | PVP01_1301700 | A0A1G4H2Q1 | 226 | 26.4 |
|  |  | *Pk*-H | PKNH_1302300 | A0A1Y3DTW3 | 226 | 26.4 |
| 7 | CYP32 | *Pf*-3D7 | PF3D7_1215200 | Q8I5Q4 | 280 | 32.3 |
|  |  | *Pv*-P01 | PVP01_1434000 | A0A1G4H4X8 | 276 | 31.9 |
|  |  | *Pk*-H | PKNH_1434700 | A0A1Y3DN49 | 276 | 31.9 |
| 8 | CYP52 | *Pf*-3D7 | PF3D7_1423200 | Q8ILM0 | 446 | 52.7 |
|  |  | *Pk*-H | PKNH_1334800 | A0A1A7VCB7 | 441 | 51.5 |
| 9 | CYP72 | *Pf*-3D7 | PF3D7_0930600 | Q8I2K8 | 609 | 72.6 |
|  |  | *Pk*-H | PKNH_0729200 | A0A1Y3DRD2 | 609 | 71.2 |
| 10 | CYP81 | *Pf*-3D7 | PF3D7_0803000 | Q8IAN0 | 677 | 80.9 |
|  |  | *Pv*-P01 | PVP01_0117200 | A0A1G4GR20 | 590 | 65.3 |
|  |  | *Pk*-H | PKNH_0115900 | A0A1Y3DZD1 | 597 | 67.6 |
| 11 | CYP87 | *Pf*-3D7 | PF3D7_0510200 | Q8I402 | 747 | 87.0 |
|  |  | *Pv*-P01 | PVP01_1023800 | A0A1G4GYV3 | 731 | 82.7 |
|  |  | *Pk*-H | PKNH_1023400 | A0A1Y3DWD7 | 723 | 82.7 |

**Supplementary Table 2**: Comparison of CsA interacting residues within 4Å and 4-5Å (in grey) in *hs*CyPs and *Plasmodium*CyPs. The numbering corresponding to *hs*CyPA is provided for reference.

| *hs*PPIA | R55 | I57 | F60 | M61 | Q63 | G72 | T73 | A101 | N102 | A103 | G104 | Q111 | F113 | W121 | L122 | K125 | H126 |
| --- | --- | --- | --- | --- | --- | --- | --- | --- | --- | --- | --- | --- | --- | --- | --- | --- | --- |
| *hs*PPIB | R | I | F | M | Q | G | T | A | N | A | G | Q | F | W | L | K | H |
| *hs*PPIC | R | I | F | M | Q | G | T | A | N | A | G | Q | F | W | L | K | H |
| *hs*PPID | R | I | F | M | Q | G | T | A | N | A | G | Q | F | H | L | K | H |
| *hs*PPIE | R | I | F | M | Q | G | T | A | N | S | G | Q | F | W | L | K | H |
| *hs*PPIF | R | I | F | M | Q | G | T | A | N | A | G | Q | F | W | L | K | H |
| *hs*PPIG | R | V | F | M | Q | G | R | A | N | R | G | Q | F | H | L | H | H |
| *hs*PPIH | R | I | F | M | Q | G | T | A | N | S | G | Q | F | W | L | K | H |
|  | | | | | | | | | | | | | | | | | |
| *p*CYP19A | R | I | F | M | Q | G | S | A | N | A | G | Q | F | W | L | K | H |
| *p*CYP19B# | R | I | F | M | Q | G | T | A | N | A | G | Q | F | W | L | K | H |
| *p*CYP19C | R | I | F | A | Q | G | K | A | N | K | G | Q | F | H | L | I/V | Y |
| *p*CYP23 | R | I | Y | I | Q | G | K | S | N | N | G | Q | F | H | L | K | H |
| *p*CYP24 | R | I | F | M | Q | G | S | A | N | T/S | G | Q | F | W | L | K | N |
| *p*CYP26 | R | V | F | M | Q | G | Y/F | C | Q | T | R | Q | F | W | L | R/K | H |
| *p*CYP32 | K | Y | I | G | V | D | K | F | N | E | G | Q | I | I | L | H | N |
| *p*CYP52$ | R | I | F | L | Q | G | L | A | N | L | N | Q | F | W | L | K | N/S |
| *p*CYP72$ | K | Q/D | K/E | N/D | V | D | E | Y/F | K/R | Y | Y | M | Y | N/E | Y | P/G | D |
| *p*CYP81 | R/K | K | K | C | K | Y | E | V | Q | V | E/G | I/V | K | T/K | Y | R/K | N |
| *p*CYP87 | R | I | F | M | Q | G | T | A | N | C | G | Q | F | W | L | K | H |

Conserved residues are in black, while those present only in Pf, Pv and Pk are shown in red, violet and green, respectively; # - Only observed in Pf; $ - Not observed in Pv

**Supplementary Table 3**: Interactions made by CsA with the human and *Plasmodium* CyPs. The residues forming hydrogen bonds and non-polar interactions are shown in bold and normal fonts, while those involved in C-H..O interactions are underlined

| *hs*CyPA | V9 | **R55** | F60 | M61 | **Q63** | G72 | A101 | **N102** | A103 | Q111 | F113 | **W121** | L122 | **H126** |
| --- | --- | --- | --- | --- | --- | --- | --- | --- | --- | --- | --- | --- | --- | --- |
| *p*CYP19A | **-** | **R62** | F67 | M68 | **Q70** | G79 | A108 | **N109** | A110 | Q118 | F120 | **W128** | L129 | **H133** |

**Supplementary Table 4**: Comparison of FK506 interacting residues in *Hs*FKBPs and *Plasmodium* FKBPs. It could be seen that *Plasmodium* FKBP35 conserves the canonical residues, while *Plasmodium* FKBP25 does not. The numbering corresponding to *hsFKBP12* is provided for reference.

| *hs*FKBP12 | Y26 | F36 | D37 | F46 | Q53 | E54 | V55 | I56 | W59 | Y82 | H87 | I90 | F99 |
| --- | --- | --- | --- | --- | --- | --- | --- | --- | --- | --- | --- | --- | --- |
| *hs*FKBP12.6 | Y | F | D | F | Q | E | V | I | F | Y | H | I | F |
| *hs*FKBP13 | Y | F | D | F | G | Q | V | I | W | Y | A | I | F |
| *hs*FKBP19 | Y | I | D | L | K | Q | V | I | L | Y | F | S | Y |
| *hs*FKBP22 | Y | F | H | I | L | E | A | L | W | Y | K | K | F |
| *hs*FKBP23 | Y | F | Y | K | G | Q | V | I | L | Y | Y | K | F |
| *hs*FKBP25 | Y | F | D | L | G | K | V | I | W | Y | Q | A | F |
| *hs*FKBP38 | L | - | - | L | C | D | V | I | L | Y | R | Y | L |
| *hs*FKBP51 | Y | F | D | F | G | Q | V | I | W | Y | S | K | F |
| *hs*FKBP52 | Y | F | D | F | G | E | V | I | W | Y | S | K | F |
| *hs*FKBP60 | Y | F | D | F | G | Q | L | I | M | Y | V | V | F |
| *hs*FKBP65 | Y | F | D | V | G | R | L | I | M | Y | L | L | F |
| *hs*FKBP133 | Y | F | D | L | G | K | V | I | W | V | V | T | F |
|  |  |  |  |  |  |  |  |  |  |  |  |  |  |
| *p*FKBP25 | Y | I | E/H | P | Y | K | H | I | I | H/Y | - | H | Y |
| *p*FKBP35 | Y | F | D | F | G | E | V | I | W | Y | C | S | F |

Conserved residues are in black, while those present only in Pf, Pv and Pk are shown in red, violet and green, respectively

**Supplementary Table 5**: Chemical diagram of all the PPIase inhibitors against *Plasmodium* FKBP35 studied till-date.

| FK506  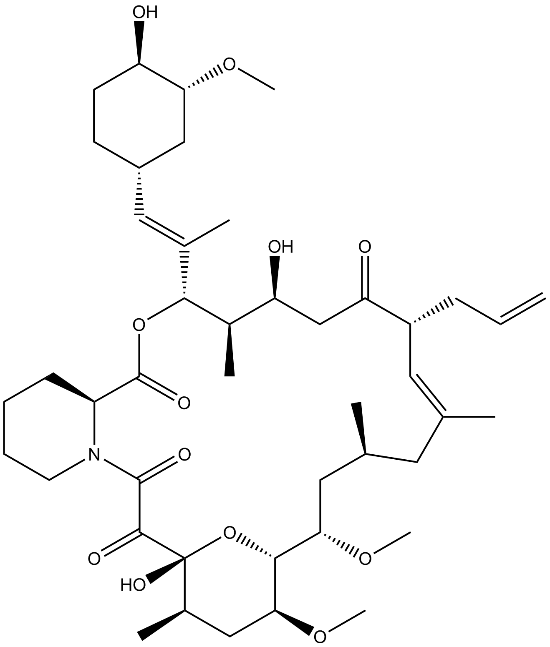 | Rapamycin  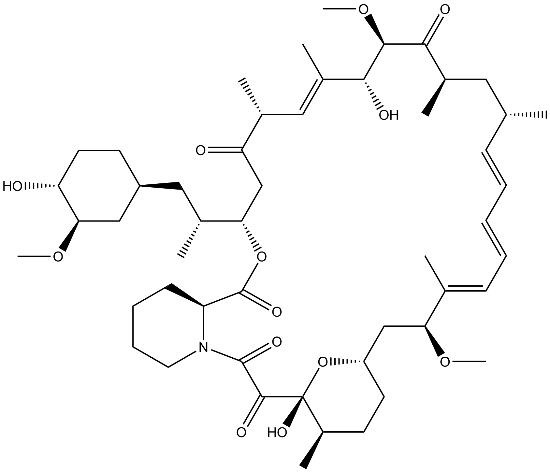 | D44  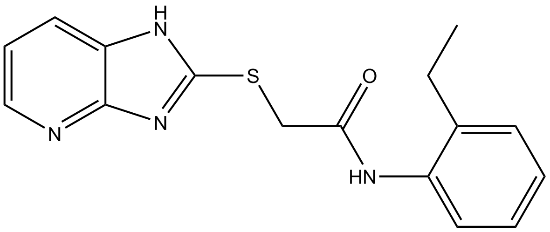 |
| --- | --- | --- |
|  |  | SRA  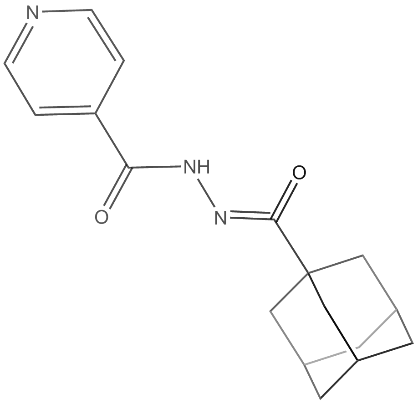 |
| [4.3.1.]ABS  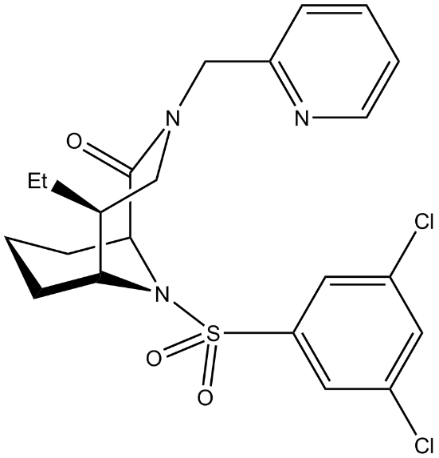 | SLF-covalent analogs  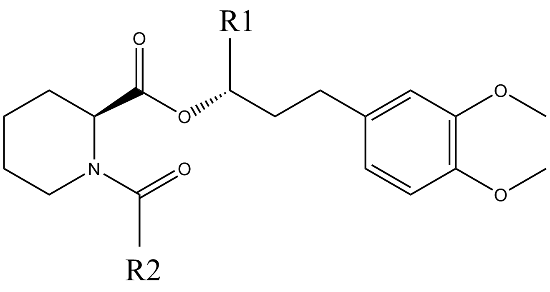 | Cyclosporin  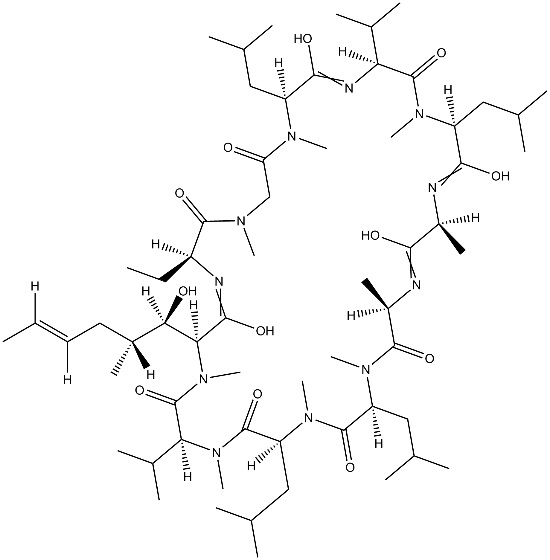 |

**Supplementary Table 6**: Interactions made by inhibitors/substrate with the human and *Plasmodium* FKBPs. The residues forming hydrogen bonds and non-polar interactions are shown in bold and normal fonts, while those involved in C-H..O interactions are underlined

| *hs*FKBP12 + FK506 | Y26 | F36 | **D37** | - | F46 | Q53 | E54 | V55 | **I56** | W59 | **-** | **Y82** | H87 | I90 | - | F99 |
| --- | --- | --- | --- | --- | --- | --- | --- | --- | --- | --- | --- | --- | --- | --- | --- | --- |
| *hs*FKBP12 + Rap | Y26 | F36 | **D37** | R42 | F46 | **Q53*** | **E54** | V55 | **I56** | W59 | A81 | **Y82** | H87 | I90 | I91 | F99 |
| *Pf*FKBD35+FK506 | Y44 | F55 | **D56** | R61 | F65 | - | E73 | V74 | **I75** | W78 | **-** | **Y101** | - | - | I110 | F118 |
| *Pv*FKBD35+FK506 | Y43 | F54 | **D55** | R60 | F64 | - | E72 | V73 | **I74** | W77 | **-** | **Y100** | - | **S108** | I109 | F117 |
| *Pf*FKBD35+ Rap | Y44 | F55 | **D56** | - | F65 | **G72** | **E73** | V74 | **I75** | W78 | **-** | **Y101** | - | - | I110 | F118 |
| *Pv*FKBD35+sALPFp | Y43 | F54 | D55 | - | F64 | - | E72 | V73 | **I74** | W77 | **-** | **Y100** | - | - | I109 | F117 |
| *Pf*FKBD35+D44 | - | - | **D56** | - | - | - | E73 | V74 | **I75** | W78 | **-** | **Y101** | C106 | - | I110 | - |
| *Pv*FKBD35+D44 | Y43 | F54 | **D55** | - | - | G71 | E72 | V73 | **I74** | W77 | **-** | **Y100** | C105 | S108 | I109 | - |
| *Pv*FKBD35+SRA | Y43 | - | D55 | - | F64 | - | - | V73 | **I74** | W77 | **-** | **Y100** | - | - | - | F117 |

* - Only H-bond
